# Unintentionally intentional: unintended effects of spinal stimulation as a platform for multi-modal neurorehabilitation after spinal cord injury

**DOI:** 10.1186/s42234-024-00144-7

**Published:** 2024-05-15

**Authors:** Gerson N. Moreno Romero, Avery R. Twyman, Maria F. Bandres, Jacob Graves McPherson

**Affiliations:** 1 Program in Physical Therapy, Washington University School of Medicine, St. Louis, MO USA; 2 Department of Anesthesiology, Washington University School of Medicine, St. Louis, MO USA; 3 Washington University Pain Center, Washington University School of Medicine, St. Louis, MO USA; 4 Program in Neurosciences, Washington University School of Medicine, St. Louis, MO USA; 5 Department of Biomedical Engineering, Washington University in St. Louis, St. Louis, MO USA

**Keywords:** Spinal cord injury, Spinal stimulation, Rehabilitation, Neuromodulation, Neural engineering, Bioelectronic medicine

## Abstract

Electrical stimulation of spinal neurons has emerged as a valuable tool to enhance rehabilitation after spinal cord injury. In separate parameterizations, it has shown promise for improving voluntary movement, reducing symptoms of autonomic dysreflexia, improving functions mediated by muscles of the pelvic floor (e.g., bowel, bladder, and sexual function), reducing spasms and spasticity, and decreasing neuropathic pain, among others. This diverse set of actions is related both to the density of sensorimotor neural networks in the spinal cord and to the intrinsic ability of electrical stimulation to modulate neural transmission in multiple spinal networks simultaneously. It also suggests that certain spinal stimulation parameterizations may be capable of providing multi-modal therapeutic benefits, which would directly address the complex, multi-faceted rehabilitation goals of people living with spinal cord injury. This review is intended to identify and characterize reports of spinal stimulation-based therapies specifically designed to provide multi-modal benefits and those that report relevant unintended effects of spinal stimulation paradigms parameterized to enhance a single consequence of spinal cord injury.

## Background and introduction

Spinal cord injury (SCI) results in a complex sequela of sensory, motor, and autonomic dysfunctions, all of which are maladaptive consequences of pathologic neural transmission in anatomically and functionally integrated networks of spinal neurons. Electrical stimulation of the spinal cord is a promising approach to enhance rehabilitation across these domains; it simultaneously modulates neural transmission across both local and distributed spinal networks. Paradoxically, however, most spinal stimulation paradigms are intended to enhance rehabilitation of only one function (e.g., voluntary movement *or* bladder function; not both).

Given the numerous, interrelated challenges faced by people living with SCI (Center NSCIS 2022; Anderson 2004; Lo et al. 2016), there is considerable motivation to develop therapies specifically intended to provide multi-modal rehabilitation benefits. Spinal stimulation appears to be uniquely capable of providing the foundation for such therapies. By defining both the core areas of consensus and the gaps in the field’s current understanding of the multi-modal effects of therapeutic spinal stimulation, it may be possible to accelerate conceptualization, development, and testing of spinal stimulation-based therapies for multi-modal rehabilitation. Towards this end, the goal of this review is to identify and characterize reports of spinal stimulation paradigms designed from the ground-up to enhance rehabilitation of multiple functions simultaneously as well as those reporting unintended effects of spinal stimulation paradigms parameterized to enhance individual functions.

## Materials and methods

### Database search parameters

The databases used for this literature search were PubMed/National Library of Medicine and Google Scholar. All searches were conducted between April and July 2023. Searches for reports of spinal stimulation-based therapies were conducted two ways: (1) directed searches that included common targets of spinal stimulation-based therapies, including bowel, bladder, sexual, autonomic, sensory, and motor functions, and (2) general searches that were not seeded with a specific SCI-related dysfunction. Searches were not limited to specific years. The following criteria were used to determine a manuscript’s suitability for inclusion:

- All studies were required to report the effects of electrical spinal stimulation.
- Spinal stimulation was defined as including epidural spinal stimulation, intraspinal microstimulation (ISMS), and transcutaneous electrical stimulation. Peripheral nerve stimulation, neuromuscular electrical stimulation, functional electrical stimulation (of muscle), and all forms of brain stimulation were excluded.
- All studies were required to report the effects of spinal stimulation on at least two sensorimotor or autonomic consequences of SCI. Studies of spinal stimulation-based therapies intended to improve a single function were permissible if they characterized and reported unintended effects on at least one other function.
- The effects of spinal stimulation were considered to be multi-modal and/or unintended only if they resulted directly from the stimulation itself. Secondary effects emerging over the course of a stimulation-enabled intervention were not considered (e.g., increased muscle mass secondary to stimulation-enabled locomotor retraining). Cognitive and affective changes were likewise excluded.
- Pre-clinical/animal studies were considered acceptable for inclusion so long as the unintended /multi-modal effects were translationally relevant to people living with SCI and not unique to the species being studied.
- Studies reporting the effects of spinal stimulation in neurologically intact animals or in people without neurological injury were also permissible if the ultimate translational application of the work was for people living with SCI (assuming all other criteria for inclusion were met and exclusion criteria were absent).
- Studies were not excluded on the grounds of injury location (e.g., cervical, thoracic, etc.), injury mechanism, time post-injury, clinical impairment level (e.g., AIS scale), or neurological category at discharge or enrollment (e.g., complete, incomplete, tetraplegia, paraplegia, etc.). However, amyotrophic lateral sclerosis, spinal muscle atrophy, and other related neurodegenerative disorders were excluded from consideration. Severe whiplash associated disorder, cervical myelopathy, and multiple sclerosis were also excluded absent radiological evidence of SCI.

## Results and discussion

In total, we identified 36 studies that fit all criteria for inclusion (Table 1). These studies ranged in publication date from 1988–2023. Additionally, we identified 2 clinical trial protocols that plan to incorporate multi-modal outcome measures (Darrow et al. 2022; Tanei et al. 2023). These protocols are not included in the figures or analyses presented below (unless explicitly stated otherwise) because trial results are not available at present.

**Table 1:** Spinal stimulation-based therapies designed for multi-modal rehabilitation and/or reporting unintended effects

**Publication date**	**First author**	**Enrollment**	**SCI classification**	**Stimulation modality**	**Stimulation location**	**Outcome measures**	**Multimodal?**	**stimulation target(s)**	**Primary unintended/mult-modal effect(s)**
1988	Barolat	16M	chronic complete and incomplete	eSCS	thoracic	OOM	N	spasms and spasticity	improved bowel, bladder, voluntary motor; no AD
1991	Katz	31M, 2F	chronic complete and incomplete	eSCS	thoracic, lumbar	OOM	N	spasticity	mixed effects on ext. sphincter dyssynergia, a/hyper-reflexia
1997	Loubser	1M	chronic incomplete	eSCS	thoracic	PRO	N	neuropathic pain	urethral spasms; urinary retention
2000	Shelyakin	25 DNR	chronic complete and incomplete	eSCS	thoracic, lumbar	PRO and OOM	Y	voluntary motor and sensation	+ sensation; + strength; + HR; - tone
2005	Ganley	2M	chronic incomplete	eSCS	thoracic, lumbar	OOM	N	voluntary motor	- 02 cost of transport; -C02; resp. exchange rate
2009	DiMarco	8M, 1F	chronic incomplete	eSCS	thoracic	PRO and OOM	N	cough	-BP; -HR; leg movements; BB unchanged
2011	Harkema	1M	chronic complete	eSCS	lumbosacral	PRO	N	voluntary motor	improved bowel, bladder, sexual, and mechanosensory perception; + spasticity
2017	Mercier	13F (rat)	acute/subacute incomplete	ISMS	cervical	OOM	N	respiration	evoked forelimb EMG
2017	Gad	1 DNR	chronic complete	tSCS	thoracic, coccxygeal	OOM	N	voluntary motor	+HR; -tone; + sensation, respiration, coordination
2017	Murray	1M	chronic incomplete	tSCS	cervicothoracic	PRO and OOM	N	voluntary motor	+ diaphoresis; - spasms, clonus; + strength; light touch unchanged; + pin prick
2018	Walter	1M	chronic complete	eSCS	lumbosacral	OOM	N	voluntary motor	- bowel prog. time; + pelvic floor tone; cardio unchanged
2018	Harkema	3M, 1F	chronic complete	eSCS	lumbar	PRO	N	cardiovascular	+ respiration; + cough
2018	Inanici	1M	chronic incomplete	tSCS	cervical	PRO and OOM	N	voluntary motor	- resid. urine vol.; + proprio; + temp. regulation
2018	DiMarco	1M	chronic incomplete	eSCS	thoracic	PRO	N	cough	motor unchanged; no bowel leakage
2018	Aslan	7M	chronic complete	eSCS	lumbosacral	OOM	Y	voluntary motor	+ BP; + HR
2018	West	1M	chronic complete	eSCS	thoracic	OOM	Y	cardiovascular	prevent ortho. hypotension; EMG unchanged
2019	DiMarco	3M	chronic complete and incomplete	eSCS	thoracic	PRO	N	cough	+ BP; + HR; bowel, bladder unchanged
2019	Sayenko	12M, 3F	chronic complete and incomplete	tSCS	lumbar	PRO and OOM	N	voluntary motor	+ spasticity; + tone; spont. voiding; bowel, sexual unchanged; no AD
2019	Nightingale	1M	chronic complete	eSCS	thoracic	OOM	Y	voluntary motor	+ V02; + MAP; + peak ventilation
2019	Darrow	2F	chronic complete	eSCS	lumbar	PRO and OOM	N	voluntary motor	mixed effects on BP, bowel, bladder, sexual
2020	Herrity	60M, 25F	chronic complete and incomplete	eSCS	lumbosacral	OOM	Y	voluntary motor	improved bladder capacity, voluntary voiding, and bladder sensation
2020	Hofstoetter	9M, 3F	chronic complete and incomplete	tSCS	lumbar	PRO and OOM	N	spasticity	improved sensation; -tone; -spasms; + hand/wrist ROM; + dexterity; bowel, bladder unchanged
2020	Wu	10M, 3F	chronic complete and incomplete	tSCS	cervical	PRO and OOM	N	voluntary motor	light headedness; nausea; discomfort; mixed HR, BP
2020	Beck	2M	chronic complete	eSCS	lumbosacral	OOM	N	voluntary motor	mixed effects on bladder, continence
2021	Inanici	4M, 2F	chronic complete and incomplete	tSCS	cervical	PRO and OOM	N	voluntary motor	- spasticity; +HR; + diaphoresis; ; + voiding; + stability
2021	Hoey	20M, 20F (rat)	chronic complete	eSCS	lumbosacral	OOM	N	bowel and bladder	+ gross motor responses
2021	DiMarco	5M	chronic complete	eSCS	thoracic	PRO	Y	cough	- bowel prog. time; no mechanical bowel methods; no incontinence; + AD
2021	Sachdeva	1M, 43M (rat)	chronic complete	tSCS	thoracic	OOM	N	autonomic dysreflexia	+ HR; - arrhythmia; + core temp; - pelvic floor EMG
2022	Kandhari	9M, 1F	acute and chronic complete	eSCS	lumbosacral	PRO	Y	voluntary motor	+ sexual; + bowel, bladder sensation; - bowel prog. time; - spasticity
2022	Herrity	6M, 1F	chronic complete	eSCS	lumbosacral	OOM	N	bladder	- BP at max bladder capacity
2022	Bandres	14M (rat)	neurologically intact	ISMS	lumbar	OOM	Y	voluntary motor and neuopathic pain	- neural transmission in spinal pain pathways
2023	Bandres	15M (rat)	chronic incomplete	ISMS	lumbar	OOM	Y	voluntary motor and neuopathic pain	- neural transmission in spinal pain pathways
2023	Bandres	15M (rat)	chronic incomplete	ISMS	lumbar	OOM	Y	voluntary motor and neuopathic pain	- neural transmission in spinal pain pathways; incr. efficacy in SCI-NP than SCI without NP
2023	McPherson	20M (rat)	chronic incomplete	ISMS	lumbar	OOM	Y	voluntary motor and neuopathic pain	- neural transmission in spinal pain pathways; incr. efficacy in SCI-NP than SCI without NP
2023	Boakye	20M, 5F	chronic complete	eSCS	lumbosacral	PRO and OOM	Y	voluntary motor and cardiovascular	general improvments along ICF domains; bladder unchanged
2023	Gorgey	1M	chronic complete	eSCS	lumbosacral	OOM	Y	voluntary motor	improved BP, HR on ortho challenge

*M* Male, *F* Female, *DNR* Did not report, *eSCS* Epidural spinal cord stimulation, *tSCS* Transcutaneous spinal stimulation, *ISMS* Intraspinal microstimulation, *PRO* Participant-reported outcome measure, *OOM* Objective outcome measure, *AD* Autonomic dysreflexia, *BP* Blood pressure, *HR* Heart rate, *MAP* Mean arterial pressure, *BB* Bowel and bladder, *SCI-NP* Spinal cord injury-related neuropathic pain, *ICF* International Classification of Functioning, Disability, and Health

**Fig. 1:**
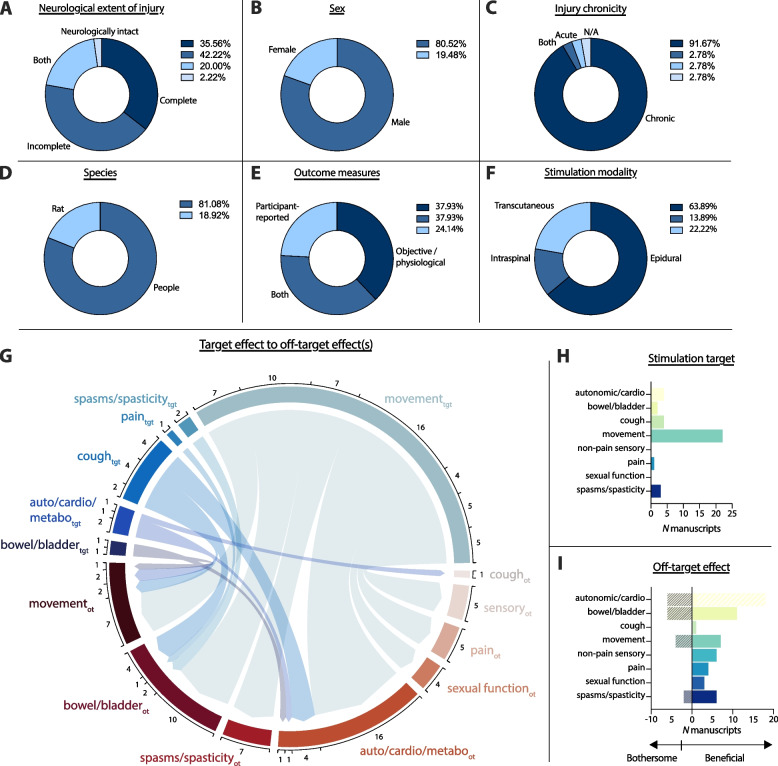
Graphical summary of spinal stimulation-based therapies designed for multi-modal rehabilitation and/or reporting unintended effects. **A**-**F** Proportion of manuscripts (*N* = 36 total) in each category. **G** Chord diagram mapping stimulation targets to intended multi-modal or unintended effect(s); tgt: primary target of stimulation; ot: multi-modal or unintended effect. Numbers represent the number of papers in each chord. Note that chords are not mutually exclusive; e.g., some manuscripts primarily targeting movement but reporting unintended effects on bowel and bladder function may also be represented in other cords, such as motor to autonomic/cardiovascular function. **H** Literal number of manuscripts (of 36) for each primary stimulation target. **I** Number of manuscripts reporting therapeutically beneficial (solid bars) vs. bothersome (shaded bars) unintended effects of stimulation

Of the 36 manuscripts reviewed below, 13 detailed spinal stimulation paradigms specifically conceptualized to afford multi-modal rehabilitation benefits (Tanei et al. 2023; Aslan et al. 2018; Harkema et al. 2018; Herrity et al. 2020; DiMarco et al. 2021; Bandres et al. 2022, 2023a, b; Kandhari et al. 2022; McPherson and Bandres 2023; Shelyakin et al. 2000; Nightingale et al. 2019; Gorgey et al. 2023; West et al. 2018). The remaining 23 manuscripts reported unintended effects of spinal stimulation paradigms designed to enhance one sensorimotor or autonomic consequence of SCI alone (Harkema et al. 2011, 2018; Barolat et al. 1988; Katz et al. 1991; Loubser 1997; DiMarco et al. 2009, 2018, 2019; Gad et al. 2017; Mercier et al. 2017; Murray and Knikou 2017; Inanici et al. 2018, 2021; Walter et al. 2018; Darrow et al. 2019; Sayenko et al. 2019; Hofstoetter et al. 2020; Beck et al. 2021; Hoey et al. 2021; Herrity et al. 2022; Ganley et al. 2005; Sachdeva et al. 2021; Wu et al. 2020). Twenty nine manuscripts reported studies of spinal stimulation in people living with SCI and 7 manuscripts utilized in vivo rat models. Ten studies of people living with SCI were *N* = 1 case reports and an additional 6 studies enrolled ≤ 5 participants. The primary characteristics of the identified manuscripts are summarized in Fig. 1, and below we synthesize the body of literature across several clinical and translational domains. In cases where synthesis across a given domain includes both human-subjects studies and animal research, the animal studies are explicitly noted. Likewise, unintended/multi-modal effects supported wholly or predominantly by case reports are explicitly noted.

### Participant characteristics

Since 2015, sensorimotor incomplete SCI has accounted for ~ 67% of all SCI in the United States, with sensorimotor complete lesions accounting for the remaining ~ 33% (Center NSCIS 2022). These proportions were not reflected in the literature, however. Indeed, 69% of manuscripts reported the effects of spinal stimulation on sensorimotor or motor *complete* SCI (of which 2 studies utilized rat models of SCI), despite only 4 of these manuscripts being published prior to 2015 (Shelyakin et al. 2000; Barolat et al. 1988; Katz et al. 1991; Harkema et al. 2011). Reports of spinal stimulation for sensorimotor incomplete SCI appeared in 19 manuscripts (53%) (Bandres et al. 2023a, b; McPherson and Bandres 2023; Shelyakin et al. 2000; Barolat et al. 1988; Katz et al. 1991; Loubser 1997; DiMarco et al. 2009, 2018, 2019; Mercier et al. 2017; Murray and Knikou 2017; Inanici et al. 2018, 2021; Sayenko et al. 2019; Hofstoetter et al. 2020; Ganley et al. 2005; Wu et al. 2020; Hubscher et al. 2018), of which 9 also included people with lesions clinically considered to be complete and 4 utilized rat models of SCI (Fig. 1A). And of the aforementioned clinical trial protocols, 1 trial intends to enroll people living with sensorimotor complete SCI (Darrow et al. 2022) and 1 trial intends to enroll people living with sensorimotor incomplete SCI (Tanei et al. 2023).

That the representation of sensorimotor incomplete SCI in the spinal stimulation literature is not reflective of its clinical prevalence is presumably related to the invasiveness of the electrode implant procedure, which introduces risk of infection and other complications. Another possibility could be that people with complete SCI are more routinely targeted for enrollment due to the perception that they have a comparative lack of alternative therapies and therefore would derive more benefit from spinal stimulation. This interpretation is supported by the predominance of studies focusing on spinal stimulation for motor rehabilitation (detailed further in subsequent sections), which, broadly, may be considered by some to be more enabling in people who would otherwise have no ability to voluntarily move. But that being said, people living with sensorimotor incomplete SCI also face a wide range of interrelated challenges unique to their particular injuries, and spinal stimulation-based therapies hold considerable promise for enhancing their quality of life, as well.

Other participant-related variables, such as sex assigned at birth and injury chronicity, were more predictable (Fig. 1B, C). People assigned male at birth and male animals represented the majority of study participants across both clinical and pre-clinical studies. Specifically, 343 males (230 people and 113 rats) were enrolled vs. 83 females (70 people and 13 rats). This distribution – 81% male – is nearly identical to that of the general population (~ 80% of people sustaining SCI were assigned male at birth) (Center NSCIS 2022).

Participants assigned female at birth were represented in 13/36 studies, with 2 additional studies reporting effects in female rats (Harkema et al. 2018; Herrity et al. 2020, 2022; Kandhari et al. 2022; Katz et al. 1991; DiMarco et al. 2009; Mercier et al. 2017; Darrow et al. 2019; Sayenko et al. 2019; Hofstoetter et al. 2020; Hoey et al. 2021; Inanici et al. 2021; Wu et al. 2020; Hubscher et al. 2018; Boakye et al. 2023). This proportion could be interpreted as a robust sample considering that females account for only one fifth of people living with SCI. Unfortunately, however, only 4 studies enrolled 5 or more female participants (Herrity et al. 2020; Mercier et al. 2017; Hoey et al. 2021; Boakye et al. 2023) (of which 2 were pre-clinical animal studies), precluding detailed subgroup analyses within or between the biological sexes. As a result, there continue to be substantial gaps both in the field’s ability to determine if and how spinal stimulation-enabled rehabilitation differs between people assigned male vs. female at birth and in defining the factors that are most useful for guiding the design of person-specific spinal stimulation-based therapies.

As aforementioned, relatively few manuscripts detailed pre-clinical/animal studies of spinal stimulation-based therapies (*N* = 7; Fig. 1D) (Bandres et al. 2022, 2023a, b; McPherson and Bandres 2023; Mercier et al. 2017; Hoey et al. 2021; Sachdeva et al. 2021). The lack of pre-clinical work may be due to the increasing investigational use of epidural spinal stimulators in clinical settings, which has enabled the impact of varying electrode montages and parameterizations to be quantified directly in the people for whom they are intended. The recent introduction of transcutaneous spinal stimulation as a potential therapeutic approach also presumably contributed (Gad et al. 2017; Murray and Knikou 2017; Inanici et al. 2018, 2021; Sayenko et al. 2019; Hofstoetter et al. 2020; Sachdeva et al. 2021). Nevertheless, given that the neural mechanisms underlying the therapeutic benefits of epidural and transcutaneous spinal stimulation remain enigmatic, it is encouraging that the shift away from pre-clinical work in this space has not been accompanied by a shift towards subjective, participant-reported outcome measures. Indeed, 25 (of 29) studies involving people living with SCI incorporated objective outcome measures centered about direct measurement of physiological variables (Darrow et al. 2019, 2022; Aslan et al. 2018; Herrity et al. 2020, 2022; Shelyakin et al. 2000; Gorgey et al. 2023; West et al. 2018; Barolat et al. 1988; Katz et al. 1991; DiMarco et al. 2009, 2019; Gad et al. 2017; Murray and Knikou 2017; Inanici et al. 2018, 2021; Walter et al. 2018; Sayenko et al. 2019; Hofstoetter et al. 2020; Beck et al. 2021; Ganley et al. 2005; Sachdeva et al. 2021; Wu et al. 2020; Hubscher et al. 2018; Boakye et al. 2023).

### Stimulation modality and location

The majority of manuscripts reported studies of epidural spinal stimulation (*N* = 22 total, including 1 rat study; Fig. 1F). Transcutaneous spinal stimulation and ISMS were represented at similar levels (tSCS: *N* = 8, including 1 rat study; ISMS: *N* = 5, all rats; Fig. 1F), although the former was studied overwhelmingly in people living with SCI whereas the latter was studied exclusively in rats. This distinction is not surprising given that inter-species anatomical differences limit the translational relevance of rat models of transcutaneous spinal stimulation and the fact that ISMS systems for clinical use have yet to gain regulatory approval.

It was somewhat counterintuitive to find ISMS amongst the stimulation modalities reporting multi-modal effects. It has traditionally been assumed that the modulatory actions of ISMS are confined to a relatively small volume surrounding the stimulation site, particularly at the sub-motor-threshold current intensities used in the studies surveyed here. However, even short bouts of ISMS delivered to the spinal motor pools appear to exert robust anti-nociceptive effects in rat models of chronic SCI-related neuropathic pain (SCI-NP) (Bandres et al. 2022, 2023a, b; McPherson and Bandres 2023). This unexpected finding is of considerable translational relevance, as ISMS also appears to confer several advantages over less focal modalities of stimulation for restoration of movement. For example, ISMS within the motor pools preserves natural motor unit recruitment order, leading to smooth, fatigue resistant contractions (Bamford et al. 2005). ISMS within the motor pools also promotes recruitment of synergistic muscle groups, facilitating coordinated limb movements even when stimulation is delivered only at a single location (Mushahwar et al. 2000). And, because electrical current is delivered directly to its intended target, ISMS systems bypass many of the indirect, highly polysynaptic pathways engaged by epidural and transcutaneous stimulation. This enables the timing of ISMS to be more precisely synchronized relative to ongoing neural activity, making it ideally suited for use in plasticity-promoting, closed-loop stimulation paradigms (McPherson et al. 2015).

Regarding stimulation location, lumbar/lumbosacral spinal stimulation was most common (58%). Thoracic stimulation followed at 36%, then cervical/cervicothoracic stimulation (14%) and coccxygeal stimulation at 3%. (Note that some studies detailed multiple stimulation locations). For perspective, sensorimotor incomplete tetraplegia – i.e., cervical injuries – are the most common neurologic category at discharge, representing 33% of all SCI (Center NSCIS 2022). Sensorimotor complete paraplegia is next, at 23.5%, followed by sensorimotor incomplete paraplegia (18.5%) and sensorimotor complete tetraplegia (18%) (Center NSCIS 2022). Thus, while cervical injuries are most common clinically, the number of cervical spinal stimulation studies in this body of literature (*N* = 5, including 1 rat study and 2 single-participant case studies) was approximately 4-fold lower than that of lumbar/lumbosacral stimulation (*N* = 21, including 5 rat studies).

### Unintended/multi-modal effects - general

Consistent with the predominance of studies enrolling people living with motor complete SCI and those utilizing a lumbar/lumbosacral stimulation site, spinal stimulation was most often parameterized to enhance rehabilitation of standing, stepping, and walking. A comprehensive review of this topic, albeit not specifically focused on unintended/multi-modal effects, has recently been published by Hachmann and colleagues (Hachmann et al. 2021). However, restoration of voluntary locomotion is far from the only rehabilitation priority for people living with SCI. Indeed, it is often not even cited as the most important,regaining use of one’s arm(s) and hand(s), and improvements in bowel, bladder, and sexual function often rated higher, with autonomic functions and SCI-related neuropathic pain (SCI-NP) also consistently among the top priorities (Anderson 2004; Lo et al. 2016). Yet, these functions occupy a curious space in the spinal stimulation literature. Whereas bowel, bladder, and autonomic functions were the most reported unintended domains (Fig. 1G), only 2 manuscripts reported studies of spinal stimulation specifically parameterized for bowel and bladder control (Hoey et al. 2021; Herrity et al. 2022) (Fig. 1G, H). Autonomic functions were targeted in 8 studies (DiMarco et al. 2009, 2018, 2019, 2021; Mercier et al. 2017; Hoey et al. 2021; Herrity et al. 2022; Sachdeva et al. 2021), whereas SCI-NP was targeted only once (Loubser 1997), and no manuscripts detailed studies specifically intended to enhance sexual function (Fig. 1G, H). With few exceptions, however, the impact of spinal stimulation on these functions was beneficial, regardless of the intended stimulation target (Fig. 1I). Below, we discuss domain-specific unintended/multi-modal effects. All 36 manuscripts were surveyed for reports of outcomes in each domain.

### Autonomic, cardiovascular, cough, and metabolic

The most common outcomes in this domain were changes in heart rate and blood pressure associated with stimulation. Stimulation parameterized to enhance voluntary movement routinely increased heart rate between ~ 15–30% in people living with SCI (Shelyakin et al. 2000; Gad et al. 2017; Inanici et al. 2021; Wu et al. 2020), although one report also noted that a subgroup of participants experienced a ~ 20% decrease in heart rate (Wu et al. 2020). Three of these 4 studies utilized tSCS (Gad et al. 2017; Inanici et al. 2021; Wu et al. 2020), although it is unclear whether that was directly linked to the observed changes. Fewer studies reported the impact of stimulation parameterized to enhance voluntary movement on blood pressure. Of those that did, however, the results were inconsistent. In one study of lumbosacral eSCS to improve voluntary movement in people living with SCI, systolic blood pressure increased by 41% and diastolic by 38%, but only in people with SCI that also had cardiac deficits (Aslan et al. 2018). In a report of cervical transcutaneous stimulation to improve voluntary movement, 7 of 13 people with SCI experienced sustained ≥ 20% increases in mean arterial pressure while 2 of 13 people with SCI experienced sustained ≥ 20% decreases in mean arterial pressure (Wu et al. 2020). In the context of orthostatic challenge, spinal stimulation increased heart rate between ~ 10–30% while rescuing ~ 30 + mmHg (systolic) in people living with SCI when lumbar/lumbosacral eSCS was parameterized to improve voluntary movement or cardiovascular function (Aslan et al. 2018; Gorgey et al. 2023; West et al. 2018; Darrow et al. 2019). It should be noted, however, that three of these studies were case reports, and as such, it is not possible to make a clear determination of the generalizability of the observed effects.

Unsurprisingly, stimulation-associated changes in heart rate and blood pressure were frequently characterized in the context of autonomic dysreflexia. In one study using thoracic tSCS to prevent or mitigate episodes of autonomic dysreflexia, the decrease in heart rate associated with digital anorectal stimulation (a common trigger of autonomic dysreflexia) was reduced by 68% during thoracic transcutaneous spinal stimulation, coupled with 82% and 65% reductions in systolic and diastolic blood pressure, respectively (*N* = 1 person living with SCI) (Sachdeva et al. 2021). Four studies reported stimulation-associated changes in heart rate and blood pressure consistent with the onset of autonomic dysreflexia, all of which utilized thoracic epidural stimulation parameterized to restore cough in people living with SCI (DiMarco et al. 2009, 2018, 2019, 2021). These episodes were marked by ~ 20% decreases in heart rate accompanied by 50% and 25% increases in systolic and diastolic blood pressure, respectively (DiMarco et al. 2009). All episodes of autonomic dysreflexia were considered asymptomatic and abated with continued use of the stimulator over the course of a multi-session intervention. One report of cervical transcutaneous stimulation also cited sustained (albeit asymptomatic) elevations or reductions in heart rate and mean arterial pressure in people living with SCI (~ 20% each), but did not state whether any participants experienced simultaneous decreases in heart rate and increases in blood pressure (Wu et al. 2020). Two studies also explicitly noted that stimulation did not cause autonomic dysreflexia in people living with SCI (thoracic eSCS for spasms and spasticity and lumbar tSCS for voluntary movement) (Barolat et al. 1988; Sayenko et al. 2019), and one study noted a 20% reduction in blood pressure at maximum bladder capacity with lumbosacral eSCS parameterized to improve bladder function in people living with SCI (Herrity et al. 2022).

Other unintended/multi-modal effects in this domain included respiratory function, thermoregulation and diaphoresis, and cough. Four studies noted improved respiratory function associated with stimulation, including a 25–50% reduction in oxygen cost of transport and reduced respiratory exchange rate (*N* = 2 participants; thoracolumbar eSCS for voluntary movement) (Ganley et al. 2005), a 15–26% increase in V0_2_ and peak ventilation (*N* = 1 participant,thoracic eSCS for voluntary movement) (Nightingale et al. 2019), and qualitative reports of an increased ability to breathe (*N* = 4 participants, lumbar eSCS for cardiovascular function,1 participant, thoracic tSCS for voluntary movement) (Harkema et al. 2018; Gad et al. 2017). One study noted a worsening of respiratory function in people living with SCI, ostensibly not due to the stimulation per se, but rather discomfort associated with the anterior and posterior cervical placement of the tSCS electrode leads (Wu et al. 2020). Five studies reported qualitative improvements in thermoregulation and/or diaphoresis (all in people living with SCI) (Harkema et al. 2011; Murray and Knikou 2017; Inanici et al. 2018, 2021; Sayenko et al. 2019), with only 1 study – thoracic tSCS to prevent or mitigate autonomic dysreflexia in rats with chronic complete SCI (Sachdeva et al. 2021) – reporting an increase in core temperature during stimulation. Improved ability to cough was explicitly noted as an unintended effect in one study, which utilized thoracic eSCS to regulate blood pressure in people living with SCI (Harkema et al. 2018). Additionally, 1 study noted that lumbar eSCS did not impact autonomic or cardiac function (*N* = 1) (Walter et al. 2018) and 2 studies noted general participant-reported benefits in this domain (Harkema et al. 2018; Boakye et al. 2023). Additional effects of spinal stimulation on autonomic functions (including but not limited to unintended/multi-modal effects) can be found in a comprehensive recent review by Flett, Garcia, and Cowley (Flett et al. 2022).

### Bowel, bladder, and sexual function

The unintended effects of spinal stimulation on bowel and/or bladder function were variable. Improved function was noted in 11 studies (Herrity et al. 2020; DiMarco et al. 2021; Kandhari et al. 2022; Barolat et al. 1988; Katz et al. 1991; Harkema et al. 2011; Inanici et al. 2018, 2021; Walter et al. 2018; Darrow et al. 2019; Beck et al. 2021), worsened function was reported in 6 studies (Katz et al. 1991; Loubser 1997; DiMarco et al. 2009; Darrow et al. 2019; Sayenko et al. 2019; Beck et al. 2021), and 7 studies reported that stimulation did not impact bowel and/or bladder function (Katz et al. 1991; DiMarco et al. 2009, 2018, 2019; Sayenko et al. 2019; Hofstoetter et al. 2020; Boakye et al. 2023), all in people living with SCI. Opposing effects on bowel and/or bladder function were also noted within individual studies (Katz et al. 1991; Darrow et al. 2019; Sayenko et al. 2019; Beck et al. 2021). Specific beneficial effects of stimulation on bladder function in people living with SCI included improved detrusor external sphincter dyssynergia and a/hyper-reflexia (although only in 2 of 23 people,thoracolumbar eSCS for spasticity) (Katz et al. 1991), emptying without a catheter (all lumbar/lumbosacral eSCS for voluntary movement) (Herrity et al. 2020; Harkema et al. 2011; Darrow et al. 2019), a ~ 43% reduction in residual urine volume (*N* = 1 participant,cervical tSCS for voluntary movement) (Inanici et al. 2018), improved continence (lumbar eSCS for voluntary movement and thoracic eSCS for cough) (DiMarco et al. 2021; Darrow et al. 2019), improved bladder capacity (lumbosacral eSCS for voluntary movement and autonomic functions) (Herrity et al. 2020), improved sensation of bladder fullness (lumbosacral eSCS for voluntary movement and autonomic functions) (Herrity et al. 2020; Kandhari et al. 2022), and reduced blood pressure at maximum bladder capacity (lumbosacral eSCS for bladder function) (Herrity et al. 2022). Instances of worsened bladder function in people living with SCI included urethral spasms resulting in urinary retention and recurrent urinary tract infections (*N* = 1 participant,thoracic eSCS for SCI-related neuropathic pain) (Loubser 1997), worsened detrusor external sphincter dyssynergia and a/hyper-reflexia (in 4/23 participants,thoracolumbar eSCS for spasticity) (Katz et al. 1991), spontaneous voiding (in 3/15 participants,lumbar tSCS for voluntary movement) (Sayenko et al. 2019), and a shift from a compliant underactive bladder to an overactive, poorly compliant bladder with sustained pressure during filling in 1 individual living with SCI yet a shift from an overactive to an underactive bladder with no change in compliance in another individual living with SCI (lumbosacral eSCS for voluntary movement) (Beck et al. 2021). Also variable were the intended stimulation targets that resulted in worsened bladder function, including spasticity, SCI-NP, cough, standing/postural control, voluntary lower limb movements, and voluntary upper limb movements.

By comparison, the unintended effects of spinal stimulation on bowel function were more uniformly positive in people living with SCI. Indeed, only 1 study noted worsened bowel function (Darrow et al. 2019),specifically, a shift in Neurogenic Bowel Dysfunction Score from moderate to severe with low-thoracic eSCS parameterized to improve both movement and autonomic functions (*N* = 1 participant; although it was noted that this individual’s bowel program duration still decreased from 90 to 30 min) (Darrow et al. 2019). To this latter point, the unintended effect of spinal stimulation on bowel program duration was particularly striking, with studies reporting 55–85% reductions in duration across studies of thoracic and lumbar eSCS targeting movement or cough (DiMarco et al. 2021; Walter et al. 2018; Darrow et al. 2019). Other beneficial bowel-related effects included improved regularity (*N* = 1 participant,lumbosacral eSCS for voluntary movement) (Harkema et al. 2011), increased external anal sphincter and pelvic floor muscle tone (*N* = 1 participant,lumbosacral eSCS for voluntary movement) (Walter et al. 2018), and elimination of mechanical methods for bowel management (*N* = 4/5 participants,thoracic eSCS for cough) (DiMarco et al. 2021). Additionally, 5 studies reported that stimulation was not associated with changes in bowel function (Barolat et al. 1988; DiMarco et al. 2009, 2018, 2019; Hofstoetter et al. 2020).

Sexual function was reported in 4 studies, all of which were conducted in people living with SCI (Kandhari et al. 2022; Harkema et al. 2011; Darrow et al. 2019; Sayenko et al. 2019). Of these, reports of improved sexual function were noted in 3 studies (Kandhari et al. 2022; Harkema et al. 2011; Darrow et al. 2019), all of which utilized eSCS and either a low-thoracic/thoracolumbar (Kandhari et al. 2022; Darrow et al. 2019) or lumbosacral (Harkema et al. 2011) electrode placement. Beneficial unintended effects on sexual function included improved arousal and ability to achieve and maintain reflexive and/or psychogenic erections (*N* = 10 and *N* = 1, respectively) (Kandhari et al. 2022; Harkema et al. 2011) and to achieve orgasm during or immediately following stimulation (*N* = 1 male participant and *N* = 1 female participant) (Harkema et al. 2011; Darrow et al. 2019). Two of the 4 studies reporting sexual function noted that participants experienced no changes in sexual function associated with stimulation (Darrow et al. 2019; Sayenko et al. 2019), including all participants (Lo et al. 2016; Bandres et al. 2023b) in a study of thoracolumbar tSCS to enable standing (Sayenko et al. 2019) and 1 of 2 participants in a multi-modal study of low-thoracic eSCS to enhance movement and autonomic functions (Darrow et al. 2019).

### Movement, muscle tone, spasms, and spasticity

The majority of unintended/multi-modal effects on movement were positive. They included reports of increased strength and/or EMG activity in functionally relevant muscles as well as reports of improved coordination and/or voluntary movement ability in people living with SCI (Shelyakin et al. 2000; Barolat et al. 1988; Gad et al. 2017; Murray and Knikou 2017; Inanici et al. 2018, 2021; Hofstoetter et al. 2020). Four studies (2 rat studies and 2 studies in people with SCI) reported deleterious movement-related effects, all of which included unintentional recruitment of non-functionally related muscles (DiMarco et al. 2009; Mercier et al. 2017; Sayenko et al. 2019; Hoey et al. 2021). Of these studies, there was no consistency between the intended targets of stimulation or the stimulation location: one study utilized thoracic epidural stimulation to restore cough in people living with sensorimotor incomplete SCI (DiMarco et al. 2009),another used cervical ISMS to enhance diaphragm function in rat models of acute/subacute SCI (Mercier et al. 2017),one study used transcutaneous lumbar stimulation to facilitate standing in people with chronic sensorimotor complete SCI (Sayenko et al. 2019),and the remaining report came from lumbosacral epidural stimulation to enhance bowel and bladder function in rats with chronic sensorimotor complete SCI (Hoey et al. 2021). Additionally, 3 studies of people living with SCI specifically noted that stimulation did *not* result in unintended effects on movement, of which 1 study used epidural stimulation to target cardiac function (West et al. 2018) and two used epidural stimulation to target cough (DiMarco et al. 2018, 2019).

Stimulation-associated changes in spasticity, spasms, and/or muscle tone were reported in 8 studies, all in people living with SCI (Kandhari et al. 2022; Shelyakin et al. 2000; Harkema et al. 2011; Gad et al. 2017; Murray and Knikou 2017; Sayenko et al. 2019; Hofstoetter et al. 2020; Inanici et al. 2021). Of these, 6 reported improved hyperreflexia (although 2 studies included only 1 participant each) (Kandhari et al. 2022; Shelyakin et al. 2000; Gad et al. 2017; Murray and Knikou 2017; Hofstoetter et al. 2020; Inanici et al. 2021) compared to only 2 studies that reported its exacerbation (Harkema et al. 2011; Sayenko et al. 2019) (of which one was an *N* = 1 case study). Interestingly, however, one study noted that transcutaneous stimulation of the lumbar spine reduced muscle tone both in the lower and upper limbs of people living with SCI (Hofstoetter et al. 2020), whereas another study of lumbar transcutaneous stimulation noted increased spasticity in the lower limbs of people living with SCI (Sayenko et al. 2019). Stimulation intensity was comparable between the studies, although the spasticity-reducing stimulation was delivered below motor threshold whereas the spasticity-promoting stimulation was delivered above motor threshold. Stimulation frequency and polarity also differed between studies (50 Hz biphasic reduced spasticity,15 or 30 Hz monophasic increased spasticity), as did the body position of participants at the time of stimulation (supine reduced spasticity,standing increased spasticity).

### Sensory function

The unintended/multi-modal effects of spinal stimulation on sensory acuity were either beneficial or negligible; no worsening of function was reported (although it should be noted that 2 studies of transcutaneous stimulation and 1 study of epidural stimulation reported general discomfort related to the electrodes/implant (Sayenko et al. 2019; Wu et al. 2020; Boakye et al. 2023). Five studies reported generalized improvements in sensation and/or proprioception in people living with SCI (Shelyakin et al. 2000; Harkema et al. 2011; Gad et al. 2017; Inanici et al. 2018; Hofstoetter et al. 2020), and an additional single-participant case study noted an increase in sensory acuity specifically to sharp, pin prick-like sensations (Murray and Knikou 2017). In 4 studies of rat models of chronic sensorimotor incomplete SCI, it was noted that lumbar ISMS intended to enhance voluntary movement also reduced spinal responsiveness to nociceptive mechanosensory feedback through wide dynamic range and nociceptive-specific spinal neurons (Bandres et al. 2022, 2023a, b; McPherson and Bandres 2023). One study in people living with SCI noted that stimulation did not cause or exacerbate SCI-NP (Sayenko et al. 2019), while an additional single-participant case study noted that sensory acuity was not altered for non-nociceptive cutaneous feedback (Murray and Knikou 2017).

The lack of spinal stimulation studies characterizing SCI-NP as an unintended/multi-modal effect was surprising, however. SCI-NP would seem to be a logical target for spinal stimulation considering that its prevalence is high (40–70% of people living with SCI) (Center NSCIS 2022), it is notoriously medically refractory, and epidural spinal stimulation was originally developed for (and remains FDA approved only for) alleviation of medically refractory pain. In fact, the electrode montages and parameter sets currently used to target other sensorimotor consequences of SCI (e.g., movement) still closely mirror those originally developed for pain-related applications. Presumably, the low number of studies reporting effects on SCI-NP is related to the fact that comparatively few studies enrolled people with sensorimotor incomplete SCI. But given that SCI-NP was not an exclusion criterion in any of the studies, additional efforts could be made to recruit and study people affected by this condition.

## Conclusions

One of the most intriguing findings of this review was the ability of a given stimulation paradigm to elicit fundamentally different, and in cases opposing, changes in otherwise clinically similar individuals. For example, the same stimulation parameters (intended to mitigate severe spasticity) resulted in conversion of detrusor areflexia to hyperreflexia in one person, conversion from detrusor hyperreflexia to areflexia in another, changes in the duration of detrusor contraction and external sphincter dyssynergia in others, yet affected no changes in others still (Katz et al. 1991). Analogous examples were also evident in other domains as well, including autonomic/cardiovascular, motor, and bowel and bladder. Findings such as these raise both physiological and technical questions about the mechanisms driving stimulation-induced effects and underscore the complexity of developing generalizable neuromodulatory solutions in a system as dynamic and interconnected as the spinal cord.

Given this complexity, it is therefore not surprising that the neural mechanisms underlying both therapeutically advantageous and deleterious effects of spinal stimulation remain incompletely understood. And as alluded to above, the available evidence also suggests that the mechanisms of action are not wholly conserved from person to person, even when stimulation parameters and clinical characteristics are comparable. Nevertheless, the prevailing view across all domains remains that recruitment of low-threshold sensory afferent fibers is a necessary, although presumably not sufficient, component (Bandres et al. 2023b; McPherson and Bandres 2023; Hachmann et al. 2021; Flett et al. 2022; Dorrian et al. 2023). Detailed descriptions of domain-specific mechanisms are available for neuropathic pain (Bandres et al. 2022, 2023b; Joosten and Franken 2020; Oakley and Prager 2002; Foreman and Linderoth 2012), movement (Hachmann et al. 2021; Eisdorfer et al. 2020), autonomic functions (Flett et al. 2022), spasticity (Nagel et al. 2017), and bowel and bladder function (Herrity et al. 2022; Janssen et al. 2017).

Several additional conclusions can be drawn from synthesis of this literature. First, however, it should again be reiterated that people living with SCI face numerous interrelated sensorimotor impairments (Anderson 2004; Lo et al. 2016), which are driven by pathologic patterns of neural transmission in networks of spinal neurons that are themselves highly interrelated. From the manuscripts included in this review, it is clear that electrical spinal stimulation modulates neural transmission across these networks, regardless of stimulation type (e.g., epidural, intraspinal), target, or location. Yet, of the thousands of manuscripts returned in queries of PubMed/National Library of Medicine and Google Scholar, we identified only 36 that described either a spinal stimulation paradigm specifically intended to afford multi-modal rehabilitation benefits or that reported the unintended effects of single-domain stimulation paradigms. The lack of manuscripts detailing unintended effects of stimulation was particularly unexpected, given that spinal stimulators were originally developed for alleviation of chronic pain, a condition distinct from their most common current use post-SCI (i.e., restoration of movement).

From a forward-looking perspective, this gap points to a clear direction in which the field can meaningfully grow. Namely, developing spinal stimulation paradigms with the express intent of affording multi-modal rehabilitation benefits. As an example, consider lumbar/lumbosacral stimulation. This paradigm is the most common both within the manuscripts detailed herein and across *all* spinal stimulation manuscripts for SCI-related applications (i.e., including those that did not report unintended effects). Given the density of sensorimotor functions mediated by lumbar and lumbosacral spinal networks, pre-clinical research and clinical trials alike would be well-positioned to interrogate its concurrent modulatory actions on voluntary motor output, bowel, bladder and sexual functions, SCI-NP, spasms, and spasticity. To motivate this point further, it is worth noting that, of the papers reviewed here, stimulation intended to enhance voluntary movement – primarily delivered at lumbar/lumbosacral sites – had unintended/multi-modal effects on every other domain considered.

The notion of purposefully engineering stimulation paradigms to afford multi-modal benefits also raises important questions pertaining to study design and how stimulation parameters are selected (i.e., current intensity, frequency, location, etc.). Except for studies specifically intended to facilitate movement, stimulation parameters are generally established with the criterion that stimulation should *not* impact motor function. While this design constraint is logical for specific use-cases – it is not difficult to envision why involuntary locomotion would be problematic for a stimulation paradigm intended to increase bladder voiding efficacy, for example – it represents something a lost opportunity in the broader context of SCI neurorehabilitation. And in many cases, it may also be unnecessary. Indeed, the unintended/multi-modal effects chronicled here were overwhelmingly beneficial (Fig. 1I). As a result, envisioning the potential implications of releasing this de facto sub-motor-threshold constraint when considering the spinal stimulation literature writ large could be a fruitful way to conceptualize new approaches to address the multi-faceted lives and priorities of people living with SCI.

As clinical trials of spinal stimulation-based rehabilitation interventions become more numerous, it will also become possible to expand the scope of what is meant by ‘multi-modal’ rehabilitation. To seed the present literature review, we used this phrase in a strict sense that encompassed only modulatory actions resulting directly from the stimulation itself. Presumably, however, many unintended effects of spinal stimulation emerge over time as a consequence of participation in stimulation-enabled rehabilitation programs that would have otherwise not been possible. Vignettes of such longer-term changes can be found in the literature, ranging from the relatively expected (e.g., changes in lean muscle mass secondary to stimulation-enabled locomotor retraining) to the unexpected (systemic immunological changes (Bloom et al. 2020). The neurorehabilitation and neural engineering communities would benefit substantially by inclusion into ongoing and future clinical trials of outcome measures designed to capture these broader multi-modal effects.

As a final consideration, we return to the notion of representation in study design. It was encouraging that some studies enrolled people living with sensorimotor incomplete SCI and/or people assigned female at birth. However, there remains considerable work to be done in this space. For example, the proportion of enrollees with sensorimotor incomplete SCI was considerably lower than the clinical prevalence of such injuries, omitting a large group of people who stand to benefit from such approaches. While valid concerns remain about the risk of complications from stimulator implantation (e.g., infection), there is considerable potential for the technology and approach to reach a point of maturity supportive of expanding the participant pool.

In the 36 years that have elapsed since publication of the first spinal stimulation study to report unintended effects on domains therapeutically relevant to people living with SCI, one study per year (on average) has been published on this topic. But based on the promising findings of this review, the central question now appears not to be *if* spinal stimulation can provide multi-modal rehabilitation benefits but rather when will the field begin to routinely develop stimulation paradigms that capitalize on this incredible potential.

## Data Availability

All data were extracted from manuscripts indexed in PubMed/National Library of Medicine and/or Google Scholar.
